# Discriminating HFrEF vs HFpEF from chest radiographs: Mitigating demographic performance gaps via augmentation and multimodal fusion

**DOI:** 10.1371/journal.pdig.0001467

**Published:** 2026-06-12

**Authors:** Han-Jay Shu, Chao-Ju Chen, Liam G. McCoy, Ryan Wang, Po-Chih Kuo, Leo Anthony Celi

**Affiliations:** 1 Department of Computer Science, National Tsing Hua University, Hsinchu, Taiwan; 2 Faculty of Medicine and Dentistry, University of Alberta, Edmonton, Canada; 3 Laboratory for Computational Physiology, Massachusetts Institute of Technology, Cambridge, Massachusetts, United States of America; 4 Division of Pulmonary Critical Care and Sleep Medicine, Beth Israel Deaconess Medical Center, Boston, Massachusetts, United States of America; 5 Department of Biostatistics, Harvard T.H. Chan School of Public Health, Boston, Massachusetts, United States of America; Durham University, UNITED KINGDOM OF GREAT BRITAIN AND NORTHERN IRELAND

## Abstract

Deep learning models applied to chest radiography have shown promise for cardiac phenotyping, yet concerns remain regarding demographic performance disparities and deployment robustness. In this proof-of-concept study, we present a fairness-centered evaluation of chest X-ray–based differentiation between heart failure with reduced ejection fraction (HFrEF) and heart failure with preserved ejection fraction (HFpEF) using publicly available data. We constructed a cohort from MIMIC-CXR linked to MIMIC-IV ICD-10 phenotypes, restricted to cases with radiographic pulmonary edema, and evaluated image-only modeling, data augmentation, and a lightweight multimodal fusion strategy incorporating demographic and comorbidity information. A DenseNet121 image-only model achieved modest discrimination (AUC = 0.61–0.64) and exhibited measurable performance gaps across race, age, and sex subgroups. Standard data augmentation improved overall performance and reduced several subgroup disparities. Multimodal fusion further enhanced discrimination (AUC = 0.76) and reduced the largest observed demographic AUC gap by up to 83% (relative reduction). Threshold-level subgroup metrics, including sensitivity and specificity, together with calibration analyses, demonstrated more balanced error profiles across demographic groups at clinically relevant operating points. These findings highlight that simple, reproducible interventions can substantially improve both performance and equity in chest X-ray–based heart failure phenotyping on public datasets.

## Author summary

We developed this proof-of-concept study because AI models built on chest radiographs may exhibit demographic performance disparities that could undermine equitable deployment, even when overall discrimination appears acceptable. Using publicly available data, we constructed a cohort from MIMIC-CXR linked to MIMIC-IV ICD-10 heart failure phenotypes and restricted analyses to cases with radiographic pulmonary edema, which enriches for acute decompensated presentations. We evaluated an image-only DenseNet121 model, standard data augmentation, and a lightweight multimodal fusion strategy that incorporates demographic and comorbidity information. We found that the image-only model achieved modest discrimination (AUC 0.61–0.64) and showed measurable gaps across race, age, and sex subgroups. Data augmentation improved overall performance and reduced several subgroup disparities, while multimodal fusion further improved discrimination (AUC 0.76) and substantially narrowed the largest demographic AUC gap. At clinically relevant operating points, threshold-level subgroup metrics (sensitivity, specificity, and predictive values) and calibration analyses suggested more balanced error profiles across demographic groups. These results indicate that simple, reproducible interventions can improve both performance and equity on public datasets, but broader validation using echocardiography-grounded labels and multi-institutional cohorts is needed, particularly for chronic or outpatient settings beyond acute presentations.

## Introduction

Heart failure (HF) affects approximately 2% of adults worldwide and remains a leading driver of hospitalisation and premature mortality [1]. Two major pathophysiological phenotypes are recognised: HF with reduced ejection fraction (HFrEF) and HF with preserved ejection fraction (HFpEF). Because pharmacologic and device therapies diverge substantially between these phenotypes [2], early and accurate differentiation is clinically important. Misclassification can lead to inappropriate treatment selection and missed opportunities for timely interventions. The current reference test is transthoracic (occasionally trans-oesophageal) echocardiography, which quantifies left-ventricular ejection fraction (LVEF). In clinical guidelines, LVEF < 40% typically denotes HFrEF, whereas LVEF ≥ 50% supports HFpEF [3].

Despite its central role, access to echocardiography remains uneven, particularly in resource-constrained or rural settings, because it requires dedicated ultrasound equipment, credentialled sonographers, and specialist interpretation. Delays or lack of access can adversely affect downstream care and prognosis, motivating interest in broadly available tools that can support early triage and decision-making. In contrast, plain chest radiographs (CXRs) are ubiquitous, inexpensive, and rapidly acquired. Clinicians can identify gross signs of HF on CXR—including pulmonary oedema, cardiomegaly, and vascular redistribution—but phenotype-level distinctions between HFrEF and HFpEF are often subtle and may lie below human perceptual thresholds.

Deep-learning (DL) methods have achieved strong performance across many medical imaging tasks [4–7], including CXR applications relevant to cardiac status such as cardiomegaly detection and binary screening for HF [8,9]. These advances support the broader premise that CXRs may encode latent morphological and textural cues beyond routine clinical interpretation. Related work has also suggested that AI models can infer HF-associated physiologic or hemodynamic surrogates from CXRs [10]. However, for HF phenotype separation, performance alone is not sufficient: a growing body of evidence shows that healthcare AI systems can exhibit systematic biases [11–14] and disproportionately misclassify patients from historically underserved groups [15,16], including women and racial minorities [11,17]. If unaddressed, such disparities could undermine equitable deployment, particularly in settings where alternative diagnostics are already limited.

Accordingly, we frame fairness as a first-class objective in evaluating CXR-based HF phenotype models. Rather than positioning phenotype separation from CXR as a novelty claim, we focus on a practical and reproducible question using public data: when differentiating HFrEF versus HFpEF from CXRs, how large are demographic performance gaps, and can simple interventions reduce them without relying on complex infrastructure? To study this question in a clinically meaningful context with strong radiographic signal, we construct a cohort from MIMIC-CXR linked to MIMIC-IV ICD-10 HF phenotypes and restrict analyses to cases with radiographic pulmonary oedema, enriching for acute decompensated presentations.

We evaluate three increasingly informative and reproducible approaches: an image-only DenseNet121 baseline, standard data augmentation, and a lightweight multimodal fusion model that adds demographics and comorbidity information. Because sensitive attributes can both mitigate and entrench disparities, we treat demographic inputs as an explicit design choice and discuss the associated ethical trade-offs [18]. We report overall and subgroup discrimination (AUC) alongside operating-point metrics to reflect deployment-relevant error trade-offs, and we use Grad-CAM to assess whether model attention highlights anatomically plausible regions. Together, these analyses provide a fairness-centered benchmark on public data and clarify when simple interventions can improve both performance and equity for CXR-based HF phenotype differentiation.

## Materials and methods

### Dataset

**Fig 1:**
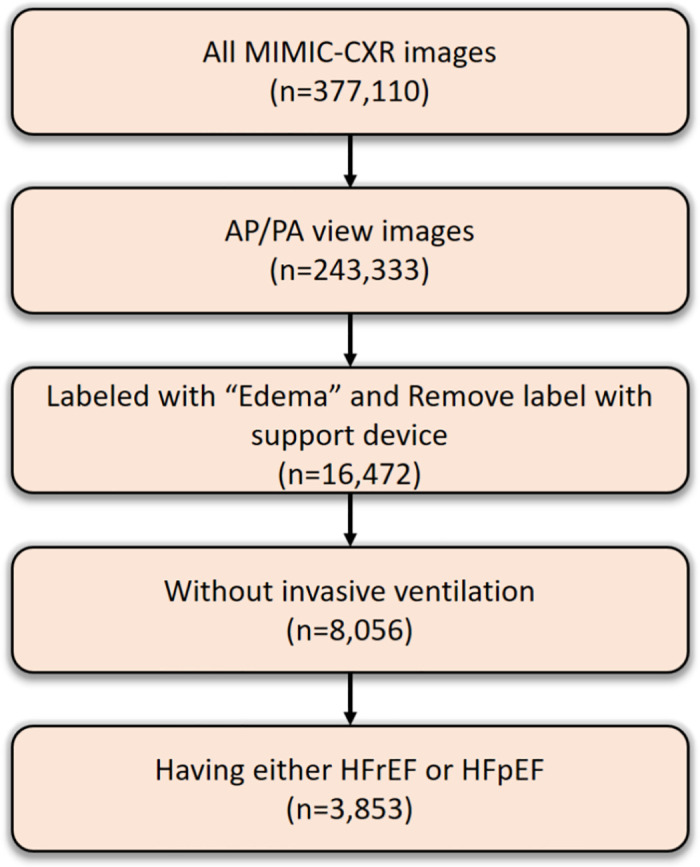
Cohort selection flow chart. The figure shows the flow chart of extracting our patient cohort and the amount of data in each step.

Fig 1 shows the cohort selection flow chart. MIMIC-CXR (MCR) [19] is a publicly available dataset of 377,110 chest radiographs, released as JPEG images in MIMIC-CXR-JPG [20,21], collected at Beth Israel Deaconess Medical Center (BIDMC) between 2011–2016. We linked MIMIC-CXR to MIMIC-IV [22] and selected adult AP/PA radiographs whose corresponding hospital admission carried an ICD-10 code for HFrEF (I50.2×) or HFpEF (I50.3×), and whose CheXpert label indicated *Edema* = 1 with *Support_Devices* = 0 (definitive labels only). To avoid within-admission correlation, we selected one *index* CXR per eligible admission (earliest AP/PA during the admission) and treated HF phenotype as an admission-level label. All dataset splits were performed at the patient level (subject_id) so that all admissions from the same patient, if present, were confined to a single split to prevent information leakage.

We linked radiographs to hospitalizations by requiring *admittime* ≤ *cxr_time* ≤ *dischtime*, where *cxr_time* was derived from DICOM *StudyDate* and *StudyTime*. In the final cohort (n = 3,853), the time from admission to the index CXR had a median of 57 hours (IQR 19–129), and the time from index CXR to discharge had a median of 148 hours (IQR 82–251). We additionally report care setting at imaging using ICU stay intervals: 2,469 (64.1%) radiographs were obtained in ward/non-ICU settings and 1,384 (35.9%) during ICU stays.

To reduce confounding from acute respiratory support, we excluded radiographs acquired during documented invasive mechanical ventilation intervals (procedureevents itemid 225792; CXR timestamp between *starttime* and *endtime*). The resulting cohort comprised 3,853 index CXRs from 3,853 admissions (1,584 HFrEF; 2,269 HFpEF). Because cohort inclusion requires radiographic pulmonary edema, analyses are enriched for acute decompensated inpatient presentations and may not generalize to stable chronic or outpatient settings.

### Model training

The dataset was split into training, validation, and testing sets using a 70%–21%–9% partition, with splitting performed at the patient level (subject_id) to prevent information leakage such that no patient appeared in more than one split. We trained a DenseNet121 architecture [23] initialized with weights pre-trained on the CheXpert dataset, and optimized all models using Adaptive Moment Estimation (Adam) with an initial learning rate of 0.001. Training was monitored on the validation set and early-stopped if the validation loss did not improve for five consecutive epochs. To assess robustness to stochastic training variation, we repeated training with five different random seeds controlling model initialization and data shuffling, and report performance aggregated across seeds. Uncertainty was quantified using non-parametric bootstrap on the held-out test set with 1000 resamples to compute 95% confidence intervals for all metrics, including AUROC, accuracy, precision, recall, specificity, and F1-score; for subgroup analyses, confidence intervals were computed within each demographic subgroup using the same bootstrap procedure.

### Data augmentation

We applied standard, label-preserving data augmentation to improve robustness to plausible acquisition variation in chest radiography (e.g., mild differences in patient positioning, rotation, and scale) and to evaluate whether such interventions can reduce demographic performance disparities [18]. Augmentations were applied *only* to the training set. For each training image, we randomly sampled augmentation parameters within pre-specified bounds and applied a random subset of transformations. We used five augmentation types: rotation, shear, translation (transformation), rescaling, and fisheye distortion. To reduce the risk of introducing anatomically implausible artifacts or altering clinically relevant structures (e.g., excessive deformation of the cardiac silhouette), all transformation magnitudes were bounded to mild ranges (see S1 Table), and we visually inspected randomly sampled augmented images during development. The impact of augmentation was evaluated in terms of overall discrimination and threshold-level performance, as well as changes in subgroup performance and disparities across race, age, and sex groups.

### Multimodal fusion

We trained a Random Forest classifier using demographic and comorbidity features (Table 1) to produce a clinical probability score $p_{\text{RF}}(y=\text{1}|x_{\text{clin}})$. The image model produced an imaging probability score $p_{\text{CXR}}(y=\text{1}|x_{\text{img}})$. We then performed lightweight late fusion by combining these modality scores to obtain a single prediction,

**Table 1:** Patient characteristics and outcomes. Abbreviations: HFpEF = Heart Failure with Preserved Ejection Fraction; HFrEF = Heart Failure with Reduced Ejection Fraction; COPD = Chronic Obstructive Pulmonary Disease; CAD = Coronary Artery Disease; CKD = Chronic Kidney Disease.

Feature	Label	HFpEF	HFrEF	Total	P-value
Age, mean (SD)	–	72.9 (13.1)	70.2 (14.6)	71.8 (13.8)	<0.001
Sex, n (%)	Female	1419 (62.5)	615 (38.8)	2034 (52.8)	<0.001
	Male	850 (37.5)	969 (61.2)	1819 (47.2)	
Ethnicity, n (%)	Asian	60 (2.6)	26 (1.6)	86 (2.2)	<0.001
	Black	384 (16.9)	198 (12.5)	582 (15.1)	
	Hispanic	82 (3.6)	79 (5.0)	161 (4.2)	
	Other	159 (7.0)	120 (7.6)	279 (7.2)	
	White	1584 (69.8)	1161 (73.3)	2745 (71.2)	
CAD, n (%)	0	1927 (84.9)	1216 (76.8)	3143 (81.6)	<0.001
	1	342 (15.1)	368 (23.2)	710 (18.4)	
COPD, n (%)	0	1506 (66.4)	1247 (78.7)	2753 (71.5)	<0.001
	1	763 (33.6)	337 (21.3)	1100 (28.5)	
Asthma, n (%)	0	1935 (85.3)	1427 (90.1)	3362 (87.3)	<0.001
	1	334 (14.7)	157 (9.9)	491 (12.7)	
CKD, n (%)	0	1872 (82.5)	1281 (80.9)	3153 (81.8)	0.211
	1	397 (17.5)	303 (19.1)	700 (18.2)	
Diabetes, n (%)	0	1238 (54.6)	810 (51.1)	2048 (53.2)	0.039
	1	1031 (45.4)	774 (48.9)	1805 (46.8)	
Malignancy, n (%)	0	2041 (90.0)	1432 (90.4)	3473 (90.1)	0.683
	1	228 (10.0)	152 (9.6)	380 (9.9)	
Dementia, n (%)	0	2027 (89.3)	1454 (91.8)	3481 (90.3)	0.013
	1	242 (10.7)	130 (8.2)	372 (9.7)	
Lung cancer, n (%)	0	2220 (97.8)	1557 (98.3)	3777 (98.0)	0.378
	1	49 (2.2)	27 (1.7)	76 (2.0)	
Rib fracture, n (%)	0	2243 (98.9)	1542 (97.3)	3785 (98.2)	0.001
	1	26 (1.1)	42 (2.7)	68 (1.8)	
Pneumothorax, n (%)	0	2249 (99.1)	1563 (98.7)	3812 (98.9)	0.245
	1	20 (0.9)	21 (1.3)	41 (1.1)	
Hypertension, n (%)	0	1452 (64.0)	1031 (65.1)	2483 (64.4)	0.506
	1	817 (36.0)	553 (34.9)	1370 (35.6)	
Imaging location	ED	131 (5.8)	77 (4.9)	208 (5.4)	0.304
	ICU	276 (12.2)	179 (11.3)	455 (11.8)	
	Ward	1862 (82.1)	1328 (83.8)	3190 (82.8)	
In-hospital mortality	0	2058 (90.7)	1413 (89.2)	3471 (90.1)	0.140
	1	211 (9.3)	171 (10.8)	382 (9.9)	


$$\begin{array}{c} p_{\text{fusion}}=\frac{p_{\text{CXR}}+p_{\text{RF}}}{\text{2}}, \end{array}$$

which enables a transparent multimodal baseline without training a joint end-to-end network. As a sensitivity analysis, we repeated the Random Forest and fusion procedure after excluding self-reported race from the clinical feature set to assess the performance–equity trade-offs of including a sensitive attribute.

### Model explanation

We implemented Grad-CAM [24] to provide qualitative insight into model decision-making by computing a class-discriminative importance map from gradients with respect to the final convolutional block of DenseNet121 and overlaying it on the input image. To assess stability, we generated Grad-CAM heatmaps for a fixed set of representative test images across the five trained seeds and report qualitatively consistent attention patterns.

### Fairness evaluation

In addition to overall model performance, we evaluated disparities across race, age, and sex groups by comparing subgroup performance for the baseline, augmentation, and multimodal models. Beyond AUROC, we report threshold-level subgroup metrics—including sensitivity, specificity, PPV, NPV, FPR, and FNR—to characterize deployment-relevant error trade-offs. Unless otherwise specified, we used a single common probability threshold of 0.5 applied identically to all demographic subgroups to enable direct comparability of error profiles across groups. We assessed calibration using the Brier score overall and by subgroup, computed from test-set predicted probabilities, without applying post-hoc recalibration. To align with established fairness notions, we additionally summarize between-group differences in TPR (sensitivity) and FPR as an equalized-odds-style disparity proxy. We quantified disparity reductions by comparing subgroup gaps across models and report relative gap reductions; uncertainty for subgroup metrics and key gap-change summaries was estimated by bootstrap resampling, consistent with the procedure described above. For gap-change inference, we bootstrap-resampled the test set, recomputed subgroup metrics and the maximum within-dimension gap for each resample, and summarized the distribution of gap changes between models using percentile 95% CIs. Furthermore, we conducted intersectional analyses post hoc. To assess whether disparity reductions were statistically meaningful, we used paired non-parametric bootstrap over admissions. For each subgroup contrast and operating point, we computed the change in gap between models (Δgap = gapB – gapA), reported 95% bootstrap CIs, and derived two-sided p-values from the bootstrap distribution.

## Results

### Image model performance

Table 2 summarizes the image-only DenseNet121 model without augmentation, which achieved modest overall discrimination (AUROC 0.627 [0.565–0.685]). A race-associated disparity was observed between Black and White patients (AUROC 0.736 [0.598–0.870] vs. 0.612 [0.538–0.683], Δ=0.124). Age-stratified performance was highest in patients aged 0–65 years (AUROC 0.731 [0.628–0.819]) and lowest in those aged 65–80 years (AUROC 0.557 [0.446–0.655]). At the common threshold of 0.5, threshold-level metrics indicated heterogeneous error profiles across subgroups (e.g., sensitivity Female 0.674 [0.608–0.741] overall; subgroup values in Table 2).

**Table 2:** Image-only DenseNet121 performance across demographic subgroups without data augmentation. We report AUROC and threshold-level metrics at a common threshold of 0.5, along with the Brier score.

Feature	Label	AUROC	Accuracy	Sensitivity	Specificity	Brier
All	–	0.627 (0.565–0.685)	0.620 (0.568–0.666)	0.674 (0.608–0.741)	0.532 (0.364–0.774)	0.237 (0.227–0.247)
Race	Black	0.736 (0.598–0.870)	0.740 (0.600–0.860)	0.759 (0.600–0.909)	0.600 (0.381–0.952)	0.212 (0.185–0.236)
	White	0.612 (0.538–0.683)	0.613 (0.547–0.671)	0.644 (0.559–0.727)	0.543 (0.279–0.842)	0.239 (0.229–0.250)
Age	0–65	0.731 (0.628–0.819)	0.725 (0.642–0.789)	0.736 (0.617–0.845)	0.686 (0.489–0.897)	0.218 (0.202–0.235)
	65–80	0.557 (0.446–0.655)	0.605 (0.500–0.694)	0.641 (0.521–0.746)	0.450 (0.232–0.867)	0.247 (0.232–0.266)
	80+	0.594 (0.484–0.691)	0.623 (0.535–0.711)	0.667 (0.550–0.774)	0.517 (0.235–0.809)	0.243 (0.227–0.260)
Sex	Female	0.595 (0.511–0.681)	0.615 (0.472–0.692)	0.779 (0.703–0.843)	0.415 (0.232–0.897)	0.229 (0.214–0.244)
	Male	0.577 (0.478–0.671)	0.605 (0.513–0.691)	0.467 (0.339–0.594)	0.625 (0.337–0.829)	0.247 (0.235–0.259)

After applying standard augmentation (Table 3), overall discrimination improved (AUROC 0.654 [0.594–0.712]) with slightly better calibration as measured by the Brier score (0.230 [0.216–0.245] vs. 0.237 [0.227–0.247]). The Black–White AUROC gap remained of similar magnitude (Black 0.777 [0.635–0.886] vs. White 0.647 [0.579–0.717], Δ=0.130). At threshold 0.5, augmentation increased overall sensitivity (0.784 [0.726–0.845]) with a corresponding decrease in specificity (0.460 [0.329–0.805]), reflecting a shift toward a more sensitive operating behavior.

**Table 3:** Image-only DenseNet121 performance across demographic subgroups with data augmentation. Metrics are computed at a common threshold of 0.5; Brier score summarizes calibration.

Feature	Label	AUROC	Accuracy	Sensitivity	Specificity	Brier
All	–	0.654 (0.594–0.712)	0.635 (0.585–0.689)	0.784 (0.726–0.845)	0.460 (0.329–0.805)	0.230 (0.216–0.245)
Race	Black	0.777 (0.635–0.886)	0.760 (0.620–0.860)	0.900 (0.769–1.000)	0.565 (0.200–1.000)	0.203 (0.166–0.240)
	White	0.647 (0.579–0.717)	0.638 (0.576–0.691)	0.755 (0.677–0.826)	0.474 (0.318–0.850)	0.230 (0.215–0.248)
Age	0–65	0.737 (0.631–0.819)	0.706 (0.633–0.780)	0.843 (0.741–0.930)	0.569 (0.360–0.981)	0.212 (0.190–0.236)
	65–80	0.574 (0.466–0.671)	0.621 (0.500–0.702)	0.776 (0.676–0.870)	0.381 (0.175–0.898)	0.246 (0.220–0.274)
	80+	0.654 (0.551–0.746)	0.667 (0.588–0.737)	0.746 (0.638–0.849)	0.500 (0.310–0.862)	0.231 (0.208–0.256)
Sex	Female	0.628 (0.546–0.714)	0.641 (0.508–0.733)	0.867 (0.804–0.921)	0.362 (0.200–0.871)	0.219 (0.197–0.241)
	Male	0.600 (0.496–0.690)	0.632 (0.507–0.711)	0.627 (0.500–0.742)	0.535 (0.267–0.897)	0.245 (0.228–0.264)

### Multimodal model performance

**Fig 2:**
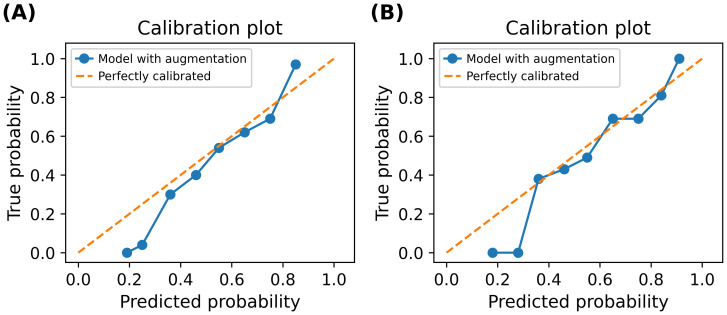
Calibration plots of the highest performing models. Shown are calibration curves comparing predicted probabilities with observed outcomes for two approaches: **(A)** DenseNet121 trained with augmentation; **(B)** a multimodal framework with pre-trained weights and augmentation.

**Fig 3:**
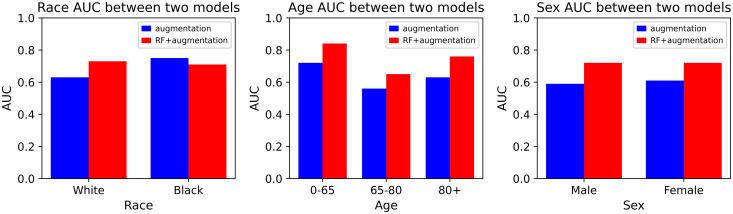
Subgroup AUCs (race, age, sex) for augmentation-only vs augmentation + Random Forest.

**Fig 4:**
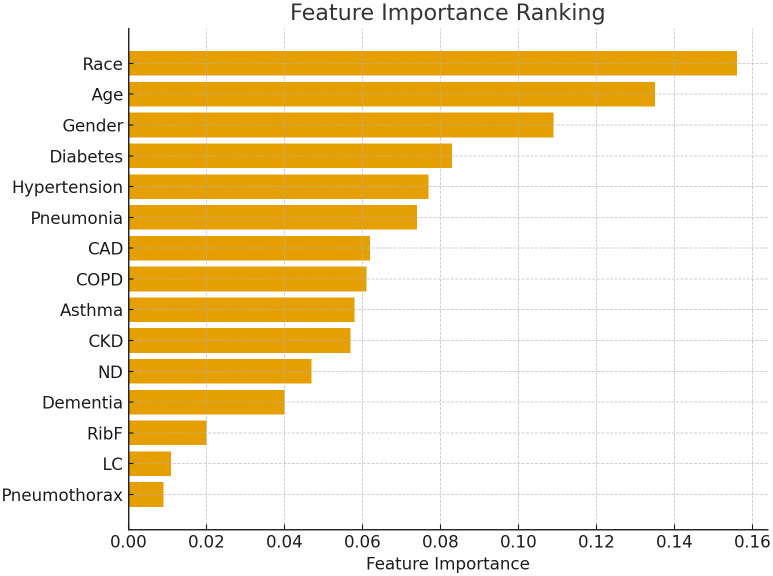
Feature importance in the random forest model. Importance scores were calculated across all demographic and comorbidity input features.

We next evaluated a lightweight multimodal fusion model that averages the image-only probability and a Random Forest probability computed from demographic and comorbidity features (Table 4). Relative to the augmentation-only image model (overall AUROC 0.654 [0.594–0.712]), multimodal fusion increased overall discrimination to AUROC 0.758 [0.708–0.806] and improved calibration (Brier 0.201 [0.184–0.217]). At the common operating threshold of 0.5, fusion shifted the error profile toward higher sensitivity (overall 0.847 [0.794–0.897]) with moderate specificity (0.557 [0.425–0.727]), consistent with the improved calibration curve (Fig 2). Across race strata, the Black–White AUROC difference decreased in magnitude from 0.130 under augmentation-only (Black 0.777 vs. White 0.647) to 0.024 under fusion (Black 0.712 vs. White 0.736), while sex-stratified AUROCs were nearly identical (Female 0.730 vs. Male 0.724). Age-related dispersion modestly widened (0–65 AUROC 0.849 vs. 65–80 AUROC 0.656), indicating that gains were not uniform across all subgroups. Fig 3 summarizes subgroup AUROCs for augmentation-only versus fusion, and Random Forest feature importances (Fig 4) suggest that demographic context contributes materially to the fusion signal.

**Table 4:** Multimodal fusion performance across demographic subgroups (with race included). Fusion averages the image-only and Random Forest probabilities. Metrics are computed at a common threshold of 0.5; Brier score summarizes calibration.

Feature	Label	AUROC	Accuracy	Sensitivity	Specificity	Brier
All	–	0.758 (0.708–0.806)	0.720 (0.671–0.764)	0.847 (0.794–0.897)	0.557 (0.425–0.727)	0.201 (0.184–0.217)
Race	Black	0.712 (0.557–0.843)	0.720 (0.560–0.840)	0.903 (0.769–1.000)	0.522 (0.250–1.000)	0.210 (0.163–0.267)
	White	0.736 (0.673–0.796)	0.712 (0.650–0.766)	0.823 (0.756–0.887)	0.554 (0.387–0.736)	0.206 (0.188–0.225)
Age	0–65	0.849 (0.772–0.917)	0.798 (0.725–0.872)	0.855 (0.755–0.942)	0.722 (0.536–0.947)	0.175 (0.150–0.200)
	65–80	0.656 (0.551–0.751)	0.685 (0.580–0.758)	0.806 (0.704–0.891)	0.410 (0.244–0.764)	0.222 (0.195–0.249)
	80+	0.769 (0.681–0.853)	0.746 (0.667–0.816)	0.892 (0.808–0.964)	0.541 (0.340–0.867)	0.202 (0.174–0.232)
Sex	Female	0.730 (0.654–0.800)	0.723 (0.549–0.795)	0.938 (0.891–0.974)	0.405 (0.231–0.972)	0.190 (0.167–0.216)
	Male	0.724 (0.650–0.803)	0.704 (0.618–0.770)	0.672 (0.548–0.781)	0.670 (0.471–0.838)	0.214 (0.193–0.233)

**Fig 5:**
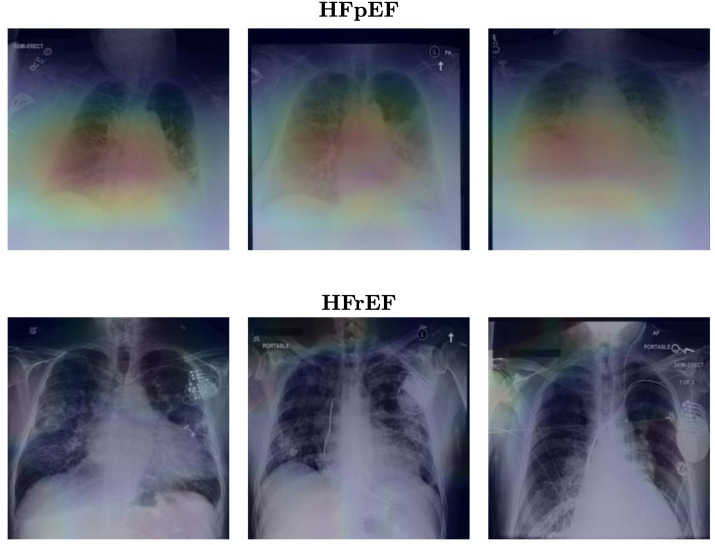
Grad-CAM examples for HFpEF and HFrEF. Representative Grad-CAM heatmaps for HFpEF (top row) and HFrEF (bottom row). Grad-CAM++ was computed with respect to the final convolutional layer (DenseNet121 denseblock4/denselayer16/conv2) targeting the positive-class logit. For stability, heatmaps were generated independently for five models trained with different random seeds and then averaged pixel-wise per image; the seed-averaged maps are shown.

To probe reliance on sensitive attributes and quantify the performance–equity trade-off, we repeated fusion after excluding self-reported race from the Random Forest inputs (Table 5). Removing race resulted in a clear degradation in overall performance and calibration, with AUROC falling from 0.758 [0.708–0.806] to 0.694 [0.637–0.746] and Brier worsening from 0.201 [0.184–0.217] to 0.226 [0.213–0.238]. Importantly, excluding race also attenuated the subgroup gap benefits observed under full fusion: the magnitude of the Black–White AUROC difference increased from |Δ|=0.024 (Black 0.712 vs. White 0.736) to |Δ|=0.058 (Black 0.740 vs. White 0.682), more than doubling the dispersion between these groups. Taken together, these results indicate that, in this cohort, race information contributed not only to higher overall discrimination but also to narrower Black–White separation in rank-based performance. Finally, Grad-CAM examples (Fig 5) continued to highlight the cardiac silhouette and pulmonary vasculature, supporting anatomically plausible attention patterns under the imaging component of the multimodal framework. Subgroup sizes and paired-bootstrap comparisons of subgroup gap changes across operating points are reported in S2 Table.

**Table 5:** Multimodal fusion sensitivity analysis (race excluded from Random Forest inputs). Metrics are computed at a common threshold of 0.5; Brier score summarizes calibration.

Feature	Label	AUROC	Accuracy	Sensitivity	Specificity	Brier
All	–	0.694 (0.637–0.746)	0.677 (0.628–0.723)	0.863 (0.814–0.911)	0.423 (0.269–0.733)	0.226 (0.213–0.238)
Race	Black	0.740 (0.596–0.869)	0.720 (0.580–0.840)	0.933 (0.821–1.000)	0.471 (0.125–1.000)	0.215 (0.178–0.251)
	White	0.682 (0.617–0.747)	0.675 (0.617–0.728)	0.841 (0.784–0.903)	0.427 (0.252–0.745)	0.226 (0.211–0.241)
Age	0–65	0.782 (0.687–0.862)	0.743 (0.661–0.826)	0.857 (0.760–0.945)	0.588 (0.357–0.922)	0.212 (0.194–0.231)
	65–80	0.579 (0.476–0.678)	0.621 (0.516–0.710)	0.819 (0.727–0.899)	0.327 (0.132–0.900)	0.242 (0.220–0.264)
	80+	0.733 (0.636–0.813)	0.719 (0.640–0.798)	0.925 (0.848–0.983)	0.426 (0.191–0.837)	0.220 (0.198–0.243)
Sex	Female	0.665 (0.582–0.747)	0.677 (0.528–0.759)	0.992 (0.975–1.000)	0.217 (0.031–0.895)	0.212 (0.192–0.234)
	Male	0.617 (0.521–0.707)	0.651 (0.559–0.724)	0.606 (0.478–0.723)	0.600 (0.407–0.864)	0.242 (0.231–0.254)

Fig 5 presents representative and averaged Grad-CAM maps for HFpEF and HFrEF predictions. Saliency concentrates around the cardiac silhouette and pulmonary vasculature, providing qualitative, illustrative evidence of anatomically plausible attention patterns.

## Discussion

Our study provides initial evidence that deep-learning models can modestly differentiate HFrEF from HFpEF using CXRs alone, demonstrating that CXRs harbor clinically relevant latent signals beyond human perceptual thresholds. The modest performance underscores the complexity of phenotype differentiation using a single imaging modality, yet offers promising initial evidence of utility, especially in settings where advanced cardiac imaging is unavailable [25]. Although clinicians cannot reliably make this distinction by eye, our findings indicate that CXRs contain latent signal capable of separating the two phenotypes. Such focus is clinically intuitive: HFrEF is often associated with ventricular dilatation, whereas HFpEF commonly involves concentric thickening that impairs relaxation [26]. The observed, albeit moderate, discrimination implies that the network captured features specific to each HF subtype. Performance gains with larger parameter counts argue against simple over-fitting. In addition, we found the data augmentation tools to be highly useful, successfully improving model performance and suggesting further generalizability.

Initial image-only models notably under-performed in male and White subgroups, echoing previous observations in medical AI literature where machine learning models inadvertently embed or amplify demographic biases [11–14,17]. These biases may arise from dataset composition, image acquisition parameters, or inherent population differences, indicating the need for detailed subgroup analyses and fairness interventions throughout model development and validation. Simple up-sampling failed to eliminate these gaps, suggesting that class imbalance alone does not fully explain the bias.

Multimodal fusion—concatenating Random Forest probabilities based on demographic and clinical features with DenseNet-derived imaging predictions—not only raised the overall model AUC but substantially narrowed performance gaps linked to race and sex. This highlights the potential for multimodal approaches to both enhance predictive accuracy and address fairness concerns, although it also raises critical questions regarding how demographic information should be responsibly integrated into clinical decision-support tools. Our race-exclusion sensitivity analysis illustrates a practical tension: removing race reduced overall discrimination and modestly increased the Black–White AUROC gap in this cohort, indicating that race may act as a proxy for unmeasured social, clinical, and access-related factors captured in structured data. However, using race in clinical prediction can also risk encoding structural inequities and may be unacceptable in some deployment contexts. We therefore present both variants to make the trade-off transparent and to encourage future work on replacing sensitive attributes with less sensitive, mechanism-linked variables.

Our study’s inclusion criteria primarily capture acute decompensated heart failure presentations, operationalized by the presence of pulmonary edema on inpatient chest radiography. Accordingly, the observed model performance should be interpreted within this acute care context, where radiographic features of congestion are expected to be prominent, and should not be taken as evidence of generalizability to stable chronic or outpatient heart failure populations. This distinction is clinically important, as imaging obtained during acute exacerbations may differ substantially from routine studies performed for chronic disease monitoring or unrelated indications, potentially altering both the available signal and error profiles. We therefore frame this work as a proof-of-concept for pulmonary-edema–positive inpatient presentations, and emphasize that validation on cohorts spanning care settings and disease acuity—ideally with echocardiography-derived ground truth and multi-institutional evaluation—is required before applying the approach beyond the acute setting.

Moreover, while our analysis leveraged a robust dataset with clear clinical diagnoses of heart failure, it did not fully differentiate between patients with chronic stable disease and those in acute exacerbation. Explicitly re-evaluating the models by first isolating acute heart failure patients, and then separately evaluating chronic presentations, will elucidate whether the latent imaging signals distinguishing HFrEF from HFpEF remain consistent or vary according to disease acuity. Clarifying this nuance is essential to refining our model and optimizing its clinical relevance across diverse patient scenarios.

Future research should prioritize refining explainability methods, aiming beyond simple visualization towards comprehensive interpretability frameworks capable of clearly elucidating clinically relevant model decisions. More sophisticated approaches, such as advanced saliency methods, concept-based explanations, or attention mechanisms explicitly correlated with known cardiac pathophysiology, should be pursued. This would enhance clinician trust and provide meaningful insights into the clinical utility and safety of the deployed algorithms.

In addition, validation studies employing larger, multicentre datasets—ideally involving diverse patient populations and standardized image acquisition protocols—are essential. Such rigorous evaluations would strengthen model robustness, improve generalizability across heterogeneous clinical contexts, and mitigate the risks associated with deployment biases. Prospective clinical trials or controlled implementation studies, embedded directly within routine clinical workflows, are further warranted to assess real-world effectiveness, usability, and integration challenges before any practical adoption of these AI-driven decision-support tools. Ultimately, the pathway from promising preliminary results to safe clinical deployment necessitates an interdisciplinary approach. Clinicians, data scientists, radiologists, and healthcare ethicists must collaboratively address the technical, clinical, and ethical dimensions of model deployment, ensuring that integration enhances, rather than complicates or biases, the delivery of high-quality patient care.

## Conclusion

This study presents a fairness-centred proof of concept for chest X-ray–based heart failure phenotyping in a pulmonary-edema–positive inpatient cohort. Rather than positioning chest radiography as a replacement for echocardiography, our primary contribution is an equity-focused evaluation showing that common deployment-oriented interventions—data augmentation and a simple multimodal fusion with demographic and comorbidity features—can improve overall discrimination (AUC 0.76) while reducing subgroup performance gaps at clinically relevant operating points. Because the cohort definition enriches for acute decompensated inpatient presentations, the findings are best interpreted within this acute care context and may not generalize to stable chronic or outpatient heart failure populations. Future work should prioritize external, multi-institutional validation with echocardiography-derived ground truth, and report robustness, calibration, and subgroup error profiles across care settings and disease acuity. With rigorous validation and transparent reporting, this work provides a public-data baseline for developing and assessing more equitable decision-support tools in resource-constrained settings.

## Supporting information

S1 Table Data augmentation parameter ranges. For augmented training samples, we decoded each image to grayscale and then applied one randomly chosen transformation from the set {scaling, shear, translation, rotation, fisheye} (via RandomChoice), followed by tensor conversion and intensity normalization.
(DOCX)

S2 Table Statistical comparison of disparity changes (paired bootstrap). We report the change in subgroup performance gaps between the baseline and improved models (∆gap = gapfusion − gaporig) with 95% non-parametric bootstrap confidence intervals and two-sided p-values. Operating points include rank-based AUROC gaps, a fixed-threshold setting (Thr = 0.5), and a sensitivity-constrained setting (Sens@0.80). *Note:* Test-set subgroup sizes for each contrast were: 80+ (n = 114) vs 0–65 (n = 109); Female (n = 195) vs Male (n = 152); Black/African American (n = 50) vs White (n = 243).
(DOCX)
