# Supplementary material for: Discriminating HFrEF vs HFpEF from chest radiographs: Mitigating demographic performance gaps via augmentation and multimodal fusion
Source: PLOS Digit Health. 2026 Jun 12;5(6):e0001467. doi: 10.1371/journal.pdig.0001467 (PMC13262823; doi:10.1371/journal.pdig.0001467)
Supplement: S1 Table — For augmented training samples, we decoded each image to grayscale and then applied one randomly chosen transformation from the set {scaling, shear, translation, rotation, fisheye} (via RandomChoice), followed by tensor conversion and intensity normalization. (DOCX) [file pdig.0001467.s001.docx]

**Supporting information**

| **Augmentation** | **Implementation (code)** | **Parameter range used** |
| --- | --- | --- |
| Scaling | RandomAffine(degrees=0, scale=(0.4, 1.0)) | Scale factor $s\in[0.4, 1.0]$ |
| Shear | RandomAffine(degrees=0, shear=np.pi/5) | Shear magnitude = $\pi/5$ (as implemented; $\approx{36}^{\circ}$) |
| Translation | RandomAffine(degrees=0, translate=(0.2, 0.2)) | Up to $\pm20\%$ of image width/height in each axis |
| Rotation | RandomAffine(degrees=20) | Rotation angle $\theta\in[-{20}^{\circ}, {20}^{\circ}]$ |
| Fisheye distortion | fish(img, distortion_coefficient=0.2) | Distortion coefficient $k=0.2$; |
|  |  | $(c_{x},c_{y})\sim\text{Uniform}\{48,\ldots,208\}$ on a $224\times224$ grid |

**S1 Table: Data augmentation parameter ranges.** For augmented training samples, we decoded each image to grayscale and then applied one randomly chosen transformation from the set {scaling, shear, translation, rotation, fisheye} (via RandomChoice), followed by tensor conversion and intensity normalization.
