# Supplementary material for: Discriminating HFrEF vs HFpEF from chest radiographs: Mitigating demographic performance gaps via augmentation and multimodal fusion
Source: PLOS Digit Health. 2026 Jun 12;5(6):e0001467. doi: 10.1371/journal.pdig.0001467 (PMC13262823; doi:10.1371/journal.pdig.0001467)
Supplement: S2 Table — We report the change in subgroup performance gaps between the baseline and improved models (∆gap = gapfusion − gaporig) with 95% non-parametric bootstrap confidence intervals and two-sided p-values. Operating points include rank-based AUROC gaps, a fixed-threshold setting (Thr = 0.5), and a sensitivity-constrained setting (Sens@0.80). Note: Test-set subgroup sizes for each contrast were: 80+ (n = 114) vs 0–65 (n = 109); Female (n = 195) vs Male (n = 152); Black/African American (n = 50) vs White (n = 243). (DOCX) [file pdig.0001467.s002.docx]

| **Group** | **Contrast** | **Metric** | **Operating point** | $\Delta$**gap (95% CI),** $p$ |
| --- | --- | --- | --- | --- |
| Age | 80+ $-$ 0–65 | AUROC gap | Rank-based | 0.055 ($-$0.080 to 0.211), 0.464 |
| Age | 80+ $-$ 0–65 | TPR gap | Sens@0.80 | 0.022 ($-$0.142 to 0.185), 0.764 |
| Age | 80+ $-$ 0–65 | FPR gap | Thr=0.5 | 0.057 ($-$0.201 to 0.308), 0.688 |
| Age | 80+ $-$ 0–65 | NPV gap | Thr=0.5 | 0.105 ($-$0.116 to 0.323), 0.332 |
| Age | 80+ $-$ 0–65 | PPV gap | Thr=0.5 | 0.003 ($-$0.138 to 0.158), 0.948 |
| Sex | Female $-$ Male | AUROC gap | Rank-based | $-$0.009 ($-$0.142 to 0.113), 0.872 |
| Sex | Female $-$ Male | TPR gap | Sens@0.80 | $-$0.069 ($-$0.218 to 0.075), 0.340 |
| Sex | Female $-$ Male | FPR gap | Thr=0.5 | 0.003 ($-$0.194 to 0.209), 0.992 |
| Sex | Female $-$ Male | NPV gap | Thr=0.5 | 0.181 ($-$0.006 to 0.381), 0.056 |
| Sex | Female $-$ Male | PPV gap | Thr=0.5 | $-$0.047 ($-$0.157 to 0.068), 0.476 |
| Race | Black/African American $-$ White | AUROC gap | Rank-based | $-$0.149 ($-$0.327 to 0.022), 0.088 |
| Race | Black/African American $-$ White | TPR gap | Sens@0.80 | $-$0.104 ($-$0.258 to 0.047), 0.196 |
| Race | Black/African American $-$ White | FPR gap | Thr=0.5 | 0.115 ($-$0.173 to 0.403), 0.468 |
| Race | Black/African American $-$ White | NPV gap | Thr=0.5 | $-$0.032 ($-$0.323 to 0.254), 0.832 |
| Race | Black/African American $-$ White | PPV gap | Thr=0.5 | $-$0.069 ($-$0.224 to 0.073), 0.332 |

**S2 Table: Statistical comparison of disparity changes (paired bootstrap).** We report the change in subgroup performance gaps between the baseline and improved models (∆gap = gapfusion − gaporig) with 95% non-parametric bootstrap confidence intervals and two-sided p-values. Operating points include rank-based AUROC gaps, a fixed-threshold setting (Thr=0.5), and a sensitivity-constrained setting ([Sens@0.80](mailto:Sens@0.80)).

*Note:* Test-set subgroup sizes for each contrast were: 80+ (n = 114) vs 0–65 (n = 109); Female (n = 195) vs Male (n = 152); Black/African American (n = 50) vs White (n = 243)
