# Supplementary material for: Discriminating HFrEF vs HFpEF from chest radiographs: Mitigating demographic performance gaps via augmentation and multimodal fusion
Source: PLOS Digit Health. 2026 Jun 12;5(6):e0001467. doi: 10.1371/journal.pdig.0001467 (PMC13262823; doi:10.1371/journal.pdig.0001467)
Supplement: Attachment — Submitted filename: Response letter.pdf [file pdig.0001467.s004.pdf]

Manuscript Title: Discriminating HFrEF vs HFpEF from chest radiographs: mitigating demographic performance gaps via augmentation and multimodal fusion

General Response: We would like to thank the Section Editor and both reviewers for their thoughtful and constructive comments. Their feedback has been invaluable in helping us strengthen the clarity, methodological rigor, and clinical framing of our manuscript. In response to these comments, we have substantially revised the manuscript to better position the study's contribution, clarify cohort construction and task boundaries, expand fairness-related evaluation, and improve transparency in both the modeling and interpretability analyses. Major revisions include:

1. Reframing the manuscript as a fairness-centered, public-data proof-of-concept evaluation of chest radiograph–based differentiation between HFrEF and HFpEF, rather than as a first demonstration that chest radiographs contain EF-related signal.

2. Clarifying the cohort definition and task boundary, including the linkage between MIMIC-CXR and MIMIC-IV, the use of ICD-10 phenotypes together with radiographic pulmonary edema, the timing of the index chest radiograph relative to admission and discharge, and the resulting enrichment for acute decompensated inpatient presentations rather than stable chronic or outpatient heart failure populations.

3. Strengthening the experimental design description by explicitly stating that all dataset splits were performed at the patient level to prevent information leakage.

4. Expanding the fairness evaluation beyond AUROC by adding threshold-level subgroup metrics, including sensitivity and specificity, together with calibration analyses using the Brier score. We additionally report an equalized-odds-style disparity proxy and use paired non-parametric bootstrap to assess the statistical uncertainty of subgroup gap changes.

5. Adding a sensitivity analysis excluding self-reported race from the multimodal fusion inputs to examine the performance–equity trade-offs associated with including a sensitive demographic variable.

6. Improving the description of data augmentation by clarifying the rationale for using mild, label-preserving transformations intended to reflect plausible acquisition variation while avoiding anatomically implausible distortions.

7. Enhancing the Grad-CAM methodology description by specifying the target layer, target output, and the across-seed averaging procedure used to assess stability.

8. Revising the Abstract, Discussion, and Conclusions to more explicitly acknowledge the modest performance of the multimodal model relative to echocardiography as the

clinical reference standard, to delimit the intended proof-of-concept scope of the study, and to emphasize the need for future validation using echocardiography-grounded labels and multi-institutional cohorts.

We have provided a detailed point-by-point response to each reviewer comment below and have revised the manuscript accordingly.

Reviewer #1

Comment 1. Summary / novelty issues
This proof of concept study uses MIMIC-CXR images linked to MIMIC-IV ICD-10 labels (I50.2× vs I50.3×, restricted to CheXpert pulmonary edema positive cases) to distinguish HFrEF from HFpEF with a DenseNet-based model, evaluates data augmentation, and shows that a simple multimodal fusion of imaging with demographics and comorbidities increases AUC from approximately 0.64 to approximately 0.75 while narrowing demographic performance gaps.

Response: We thank the reviewer for this careful summary and important framing of the study. We agree that, as originally written, the manuscript risked overstating novelty if interpreted primarily as showing for the first time that chest radiographs encode information related to ejection-fraction phenotype. In response, we substantially revised the manuscript to clarify that the principal contribution of this work is not a first demonstration of EF-related signal in chest radiographs, but rather a fairness-centered evaluation on public data examining whether simple and reproducible interventions—specifically data augmentation and lightweight multimodal fusion—can improve both predictive performance and demographic equity in this task.

Accordingly, we revised the Abstract, Introduction, Discussion, and Conclusions to reposition the manuscript as a public-data proof-of-concept study focused on subgroup disparities and mitigation strategies. We now explicitly state that the present cohort is constructed from MIMIC-CXR linked to MIMIC-IV ICD-10 phenotypes and restricted to edema-positive cases, and therefore should be interpreted as an acute inpatient, proof-of-concept setting rather than a definitive clinical EF phenotyping benchmark. We also clarify that future validation using echocardiography-grounded labels and multi-institutional cohorts is needed to assess broader clinical applicability.

Comment 2. Novelty issues
(1) The manuscript frames itself as a proof of concept showing that chest radiographs contain latent signal capable of separating HFrEF from HFpEF, and that fairness interventions such as data augmentation and multimodal fusion with demographic features (age, sex, self-reported race) can improve performance.
However, the core premise that chest radiographs encode EF-related signal has already been demonstrated at multi-institutional scale with external validation and echocardiographic

ground truth. Ueda et al. (Lancet Digital Health, 2023) trained with patient-level splits using five-fold cross-validation across several institutions and evaluated on an independent external hospital; for LVEF less than 40 percent, the external test AUC was 0.92 (95% CI 0.90 to 0.95). That work also used echo-derived labels and reported broad cardiac targets (EF, valvular disease, TR velocity), thereby establishing more strongly than the present manuscript both the presence of EF information in chest radiographs and out-of-sample generalisability (DOI: 10.1016/S2589-7500(23)00107-3). If the novelty is articulated as showing that chest radiographs can separate EF phenotypes and that augmentation or multimodality improves performance, the contribution is materially diminished by the above evidence.
(2) A differentiated contribution can be claimed by reframing fairness as a first-class study aim. Unlike many high-performing CXR-EF studies, this manuscript explicitly targets demographic subgroup gaps and investigates interventions such as augmentation and fusion with demographic and comorbidity features to narrow them. The results section and figures foreground subgroup AUCs and Random Forest feature importance, in which race, age, and sex rank highest, which indicates that demographic context materially drives the fusion gains. This line of inquiry, namely systematic fairness analysis and disparity mitigation on a public dataset, can constitute the paper's primary incremental value if it is presented clearly as such and supported with threshold-level metrics as outlined in section 3. A recommendation on positioning is to replace any first-of-its-kind framing about CXR-based EF discrimination with the following: "We provide a public-data, fairness-centred evaluation of CXR-clinical fusion for HF phenotype separation, and empirically show reduction of demographic performance gaps." The manuscript should also acknowledge the performance and external-validity advantage of echo-labelled, multi-centre work such as Ueda et al. and the clinical-utility focus of Kusunose et al., and then delineate a distinct aim that emphasises equity-oriented methodology to avoid overclaiming novelty.

Response: We thank the reviewer for this important comment and agree with the recommendation. In the revised manuscript, we have repositioned the study's contribution to emphasize fairness-oriented evaluation and disparity mitigation rather than claiming novelty based on the existence of EF-related signal in chest radiographs alone. Specifically, we now describe the work as providing a fairness-centered evaluation of chest X-ray–based HFrEF/HFpEF differentiation using publicly available data, with a focus on demographic performance gaps and the empirical effects of simple mitigation strategies.

We have also revised the text to more clearly situate our work relative to prior studies that used echo-derived labels, multi-institutional development, and external validation. In particular, we now acknowledge that studies such as Ueda et al. provide stronger evidence for out-of-sample EF prediction performance under more clinically grounded labeling schemes, whereas our study addresses a different question: how subgroup disparities manifest in a reproducible public-data setting, and whether straightforward interventions can reduce those disparities. We believe this repositioning more accurately reflects the manuscript's incremental contribution and avoids overclaiming novelty.

Comment 3. Experimental design and analysis: label definition and task boundary
Label definition and task boundary. The cohort is defined by ICD-10 codes (HFrEF and HFpEF) plus a CheXpert pulmonary edema label, which may bias toward acute congestion phenotypes rather than a pure EF phenotype; please report the timing windows among CXR, ICD, and edema labels, and discuss differences from echo-labelled tasks, for example Ueda et al.

Response: We appreciate this important observation and agree that the cohort construction should be described more explicitly. In the revised manuscript, we have clarified the task boundary and cohort definition in substantially greater detail. We now explicitly state that we linked MIMIC-CXR radiographs to MIMIC-IV hospital admissions carrying ICD-10 codes for HFrEF or HFpEF, restricted inclusion to cases with radiographic pulmonary edema, and selected one index AP/PA chest radiograph per eligible admission. We further report the temporal relationship between the index chest radiograph and hospitalization, including the median time from admission to the index CXR and from index CXR to discharge, and we provide the care-setting distribution at imaging (ICU vs non-ICU).

Importantly, we now explicitly state that, because cohort inclusion requires radiographic pulmonary edema, the resulting dataset is enriched for acute decompensated inpatient presentations and may not generalize to stable chronic or outpatient heart failure populations. We also revised the Discussion and Conclusions to distinguish this ICD-linked, edema-positive task from echo-labelled EF prediction tasks and to note that future work using echocardiography-grounded labels will be necessary for more direct clinical translation.

Comment 4. Experimental design and analysis: unit of split and potential leakage
Unit of split and potential leakage. The dataset comprises 3,853 unique admissions, and a single 70/21/9 partition is used; it is not explicit that splitting is at the patient level. Please enforce patient-level splits and report both admission-level and patient-level results.

Response: We thank the reviewer for highlighting the need for greater clarity regarding data partitioning. In the revised manuscript, we now state explicitly that all dataset splits were performed at the patient level (subject_id) to prevent information leakage, such that no patient appeared in more than one split. We also clarify that one index chest radiograph was selected per eligible admission, and that in the final cohort the number of index chest radiographs equals the number of admissions included.

Because the final analytic cohort contains one index radiograph per eligible admission and splitting was already enforced at the patient level, the principal leakage concern raised by the reviewer has been addressed in the revised Methods text through clearer reporting. We agree that patient-level partitioning is essential in this setting and have made that explicit in the Dataset and Model Training sections.

Comment 5. Experimental design and analysis: fairness evidence at decision thresholds
Current claims rely mainly on changes in AUC gaps. Please add threshold-level subgroup metrics at a common sensitivity or a common threshold, with confidence intervals, and include calibration measures such as Brier score and calibration slope and intercept overall and by subgroup.

Response: We thank the reviewer for this valuable suggestion. In the revised manuscript, we substantially expanded the fairness evaluation beyond AUROC-based subgroup comparisons. Specifically, we now report threshold-level subgroup metrics, including sensitivity and specificity, using a single common probability threshold of 0.5 applied identically across demographic subgroups to enable direct comparison of deployment-relevant error profiles. We also added calibration analysis using the Brier score, reported overall and by subgroup, without post-hoc recalibration.

To further align the analysis with established fairness concepts, we now summarize between-group differences in TPR and FPR as an equalized-odds-style disparity proxy. In addition, we quantify disparity reductions between models using paired non-parametric bootstrap over admissions, reporting 95% confidence intervals and two-sided p-values for key subgroup gap changes. These additions strengthen the rigor of the fairness analysis and make the practical error trade-offs of each modeling strategy more transparent.

Table 2: Image-only DenseNet121 performance across demographic subgroups without data augmentation. We report AUROC and threshold-level metrics at a common threshold of 0.5, along with the Brier score.

Feature	Label	AUROC	Accuracy	Sensitivity	Specificity	Brier
All	–	0.627 (0.565–0.685)	0.620 (0.568–0.666)	0.674 (0.608–0.741)	0.532 (0.364–0.774)	0.237 (0.227–0.247)
Race	Black	0.736 (0.598–0.870)	0.740 (0.600–0.860)	0.759 (0.600–0.909)	0.600 (0.381–0.952)	0.212 (0.185–0.236)
Race	White	0.612 (0.538–0.683)	0.613 (0.547–0.671)	0.644 (0.559–0.727)	0.543 (0.279–0.842)	0.239 (0.229–0.250)
Age	0–65	0.731 (0.628–0.819)	0.725 (0.642–0.789)	0.736 (0.617–0.845)	0.686 (0.489–0.897)	0.218 (0.202–0.235)
Age	65–80	0.557 (0.446–0.655)	0.605 (0.500–0.694)	0.641 (0.521–0.746)	0.450 (0.232–0.867)	0.247 (0.232–0.266)

Age	80+	0.594 (0.484–0.691)	0.623 (0.535–0.711)	0.667 (0.550–0.774)	0.517 (0.235–0.809)	0.243 (0.227–0.260)
Gender	Female	0.595 (0.511–0.681)	0.615 (0.472–0.692)	0.779 (0.703–0.843)	0.415 (0.232–0.897)	0.229 (0.214–0.244)
Gender	Male	0.577 (0.478–0.671)	0.605 (0.513–0.691)	0.467 (0.339–0.594)	0.625 (0.337–0.829)	0.247 (0.235–0.259)

Table 3: Image-only DenseNet121 performance across demographic subgroups with data augmentation. Metrics are computed at a common threshold of 0.5; Brier score summarizes calibration.

Feature	Label	AUROC	Accuracy	Sensitivity	Specificity	Brier
All	–	0.654 (0.594–0.712)	0.635 (0.585–0.689)	0.784 (0.726–0.845)	0.460 (0.329–0.805)	0.230 (0.216–0.245)
Race	Black	0.777 (0.635–0.886)	0.760 (0.620–0.860)	0.900 (0.769–1.000)	0.565 (0.200–1.000)	0.203 (0.166–0.240)
	White	0.647 (0.579–0.717)	0.638 (0.576–0.691)	0.755 (0.677–0.826)	0.474 (0.318–0.850)	0.230 (0.215–0.248)
Age	0–65	0.737 (0.631–0.819)	0.706 (0.633–0.780)	0.843 (0.741–0.930)	0.569 (0.360–0.981)	0.212 (0.190–0.236)
	65–80	0.574 (0.466–0.671)	0.621 (0.500–0.702)	0.776 (0.676–0.870)	0.381 (0.175–0.898)	0.246 (0.220–0.274)
	80+	0.654 (0.551–0.746)	0.667 (0.588–0.737)	0.746 (0.638–0.849)	0.500 (0.310–0.862)	0.231 (0.208–0.256)
Gender	Female	0.628 (0.546–0.714)	0.641 (0.508–0.733)	0.867 (0.804–0.921)	0.362 (0.200–0.871)	0.219 (0.197–0.241)
	Male	0.600 (0.496–0.690)	0.632 (0.507–0.711)	0.627 (0.500–0.742)	0.535 (0.267–0.897)	0.245 (0.228–0.264)

Table 4: Multimodal fusion performance across demographic subgroups (with race included). Fusion averages the image-only and Random Forest probabilities. Metrics are computed at a common threshold of 0.5; Brier score summarizes calibration.

Feature	Label	AUROC	Accuracy	Sensitivity	Specificity	Brier
All	–	0.758 (0.708–0.806)	0.720 (0.671–0.764)	0.847 (0.794–0.897)	0.557 (0.425–0.727)	0.201 (0.184–0.217)
Race	Black	0.712 (0.557–0.843)	0.720 (0.560–0.840)	0.903 (0.769–1.000)	0.522 (0.250–1.000)	0.210 (0.163–0.267)
	White	0.736 (0.673–0.796)	0.712 (0.650–0.766)	0.823 (0.756–0.887)	0.554 (0.387–0.736)	0.206 (0.188–0.225)
Age	0–65	0.849 (0.772–0.917)	0.798 (0.725–0.872)	0.855 (0.755–0.942)	0.722 (0.536–0.947)	0.175 (0.150–0.200)
	65–80	0.656 (0.551–0.751)	0.685 (0.580–0.758)	0.806 (0.704–0.891)	0.410 (0.244–0.764)	0.222 (0.195–0.249)
	80+	0.769 (0.681–0.853)	0.746 (0.667–0.816)	0.892 (0.808–0.964)	0.541 (0.340–0.867)	0.202 (0.174–0.232)
Gender	Female	0.730 (0.654–0.800)	0.723 (0.549–0.795)	0.938 (0.891–0.974)	0.405 (0.231–0.972)	0.190 (0.167–0.216)
	Male	0.724 (0.650–0.803)	0.704 (0.618–0.770)	0.672 (0.548–0.781)	0.670 (0.471–0.838)	0.214 (0.193–0.233)

Comment 6. Experimental design and analysis: use of sensitive features
Given that race, age, and sex dominate Random Forest feature importance, please include a sensitivity analysis that removes race from the fusion inputs and discuss the associated ethical trade-offs.

Response: We appreciate this important comment. In the revised manuscript, we added a sensitivity analysis excluding self-reported race from the clinical feature set used in the Random Forest and multimodal fusion framework. This analysis was included specifically to examine the performance–equity trade-offs associated with including a sensitive demographic attribute in the prediction pipeline.

We also revised the manuscript text to discuss this issue more explicitly from an ethical perspective. Our goal was not to argue that demographic inputs should always be included, but rather to treat them as an explicit design choice with both potential benefits and risks: such variables may help reduce measured disparities in some settings, while also raising

concerns about entrenching or normalizing differential treatment pathways. We therefore believe that reporting both the main fusion model and the race-excluded sensitivity analysis provides a more transparent basis for evaluating these trade-offs.

Table 5: Multimodal fusion sensitivity analysis (race excluded from Random Forest inputs). Metrics are computed at a common threshold of 0.5; Brier score summarizes calibration.

Feature	Label	AUROC	Accuracy	Sensitivity	Specificity	Brier
All	–	0.694 (0.637–0.746)	0.677 (0.628–0.723)	0.863 (0.814–0.911)	0.423 (0.269–0.733)	0.226 (0.213–0.238)
Race	Black	0.740 (0.596–0.869)	0.720 (0.580–0.840)	0.933 (0.821–1.000)	0.471 (0.125–1.000)	0.215 (0.178–0.251)
	White	0.682 (0.617–0.747)	0.675 (0.617–0.728)	0.841 (0.784–0.903)	0.427 (0.252–0.745)	0.226 (0.211–0.241)
Age	0–65	0.782 (0.687–0.862)	0.743 (0.661–0.826)	0.857 (0.760–0.945)	0.588 (0.357–0.922)	0.212 (0.194–0.231)
	65–80	0.579 (0.476–0.678)	0.621 (0.516–0.710)	0.819 (0.727–0.899)	0.327 (0.132–0.900)	0.242 (0.220–0.264)
	80+	0.733 (0.636–0.813)	0.719 (0.640–0.798)	0.925 (0.848–0.983)	0.426 (0.191–0.837)	0.220 (0.198–0.243)
Gender	Female	0.665 (0.582–0.747)	0.677 (0.528–0.759)	0.992 (0.975–1.000)	0.217 (0.031–0.895)	0.212 (0.192–0.234)
	Male	0.617 (0.521–0.707)	0.651 (0.559–0.724)	0.606 (0.478–0.723)	0.600 (0.407–0.864)	0.242 (0.231–0.254)

Comment 7. Minor issue: duplicate word

Duplicate word: “collected from from the Beth Israel…” → remove one “from”.

Response: We thank the reviewer for catching this typographical error. We have corrected the duplicated word in the revised manuscript.

Comment 8. Minor issue: terminology

“translate transformation” → “translation (transformation)”.

Response: We appreciate the reviewer's careful reading. We have corrected this terminology in the revised manuscript to "translation (transformation)" for consistency and clarity.

Comment 9. Minor issue: punctuation
",These biases may arise …" → remove the comma after the opening punctuation.

Response: We thank the reviewer for identifying this punctuation issue. We have corrected the sentence in the revised manuscript.

Comment 10. Minor issue: figure narration / Grad-CAM settings
Grad-CAM is shown; add a sentence on settings and stability checks, such as the averaging protocol, in the caption or the supplement.

Response: We thank the reviewer for this helpful suggestion. In the revised manuscript, we expanded the Grad-CAM description in both the main text and the figure caption to improve transparency and reproducibility. We now specify that the attribution maps were computed with respect to the final convolutional layer of DenseNet121, targeting the positive-class logit, and that for stability assessment, heatmaps were generated independently for five models trained with different random seeds and then averaged pixel-wise for each representative image.

We agree that such implementation details are important for interpreting attribution maps appropriately, and we have therefore incorporated this information directly into the manuscript.

<u>Reviewer #2</u>

Comment 1. Summary
This manuscript presents a proof-of-concept study investigating the use of deep learning models to differentiate between heart failure with reduced ejection fraction (HFrEF) and heart failure with preserved ejection fraction (HFpEF) from chest radiographs. The authors employ data augmentation and multimodal fusion with demographic features to improve model performance and address demographic performance gaps. Key findings include an AUC improvement from 0.64 to 0.75, together with reductions in race- and sex-based performance disparities. While promising, several methodological and clinical considerations warrant attention.

Response: We thank the reviewer for this thoughtful summary and constructive assessment of our work. We appreciate the recognition that the manuscript addresses an important equity-related question in AI for heart failure phenotyping. In response to the reviewer's comments, we substantially revised the manuscript to strengthen the clinical framing, clarify the cohort definition and intended scope of inference, expand the fairness evaluation beyond

AUROC-based comparisons, improve the reporting of augmentation and statistical procedures, and refine the interpretation of the model explanation analyses. We believe these revisions have materially improved the transparency and rigor of the study.

Comment 2. Major Concern – Clinical Validation and Generalizability
The cohort selection criteria focused on patients with acute HF presentations (defined by pulmonary edema on CXR and new ICD-10 codes), which may limit the model's applicability to chronic HF populations. The authors should either:
a) conduct subgroup analyses separating acute versus chronic HF cases, or
b) explicitly state this limitation in the Abstract and Conclusions with recommendations for future validation in diverse clinical settings.

Response: We thank the reviewer for this important comment and agree that the cohort construction materially affects the interpretation and generalizability of the study. In the revised manuscript, we have made this limitation substantially more explicit. We now clarify that the cohort was constructed by linking MIMIC-CXR radiographs to MIMIC-IV admissions with HFrEF or HFpEF ICD-10 phenotypes and restricting inclusion to radiographs with pulmonary edema, resulting in a dataset enriched for acute decompensated inpatient presentations rather than stable chronic or outpatient heart failure populations.

Consistent with the reviewer's recommendation, we revised the Abstract, Discussion, and Conclusion to explicitly delimit the scope of the findings to this acute-care setting and to state that the current results should not be interpreted as evidence of generalizability to chronic HF populations. We further emphasize that future work should validate these findings in broader clinical settings, including cohorts with echocardiography-derived ground truth, more diverse disease acuity, and multi-institutional external data.

Because the current study is designed as a public-data proof-of-concept rather than a comprehensive clinical validation study, we chose to strengthen this limitation statement rather than introduce a post hoc acute-versus-chronic stratification that would not be fully supported by the available labeling framework.

Comment 3. Major Concern – Modest Model Performance
The overall AUC of 0.75 for the multimodal model, while improved, remains modest for clinical decision-making. The 95% CIs for some subgroups suggest overlapping performance, raising questions about robustness. The authors should:
a) discuss the clinical implications of these AUC values in the context of existing diagnostic tools (e.g., echocardiography),
b) provide sensitivity and specificity metrics to aid interpretability, and
c) consider external validation to confirm generalizability.

Response: We appreciate this important comment and agree that the clinical interpretation of the reported performance should be framed cautiously. In the revised manuscript, we more explicitly state that the multimodal model's performance, although improved relative to the

image-only baseline, remains modest and should not be interpreted as sufficient to replace established diagnostic tools such as echocardiography. We now describe the study as a proof-of-concept, equity-focused evaluation rather than a deployment-ready clinical diagnostic system.

To improve interpretability, we expanded performance reporting beyond AUROC and now provide threshold-level metrics, including sensitivity and specificity, using a common probability threshold across demographic subgroups. We also added calibration analysis using the Brier score to provide a more deployment-relevant assessment of model behavior.

With respect to external validation, we fully agree that such evaluation would strengthen the manuscript. However, because the current cohort is built from linked MIMIC resources and is intended as a public-data proof-of-concept, we have not added a new external validation cohort in this revision. Instead, we now explicitly acknowledge this as an important limitation and identify multi-institutional validation with echocardiography-grounded labels as a key priority for future work.

Comment 4. Major Concern – Fairness Evaluation Methodology
The fairness analysis measured performance gaps but did not employ established fairness metrics such as equalized odds or demographic parity. The Results section reports disparities but lacks statistical tests for significance of gap reductions. The authors should:
a) incorporate standardized fairness metrics and statistical comparisons of disparity changes,
b) justify the focus on race, age, and sex without addressing other potential biases such as socioeconomic status, and
c) include sample sizes for subgroups to assess power limitations.

Response: We thank the reviewer for this important comment and agree that the augmentation strategy should be described more carefully. In the revised manuscript, we clarified that our augmentation pipeline was designed to use standard, label-preserving transformations intended to reflect plausible variation in chest radiograph acquisition, such as mild changes in positioning, rotation, and scale, rather than aggressive transformations aimed purely at synthetic diversity.

We now specify that augmentation parameters were sampled within pre-specified bounded ranges, and we provide the transformation magnitudes in Supplementary Table S1. We also revised the Methods section to explain why the selected transformations were chosen and to state explicitly that all transformation magnitudes were kept mild to reduce the risk of introducing anatomically implausible artifacts. In particular, we note that we sought to avoid excessive geometric distortion that could alter clinically relevant structures such as the cardiac silhouette. To further guard against this, we visually inspected randomly sampled augmented images during development.

We agree that augmentation can in principle degrade or distort medically meaningful features if applied excessively. We have therefore added text clarifying that our goal was to test

whether conservative, acquisition-plausible augmentation could improve robustness and subgroup equity without materially changing the underlying label semantics.

Supporting information

Augmentation	Implementation (code)	Parameter range used
Scaling	RandomAffine(degrees=0, scale=(0.4, 1.0))	Scale factor $s \in [0.4,\ 1.0]$
Shear	RandomAffine(degrees=0, shear=np.pi/5)	Shear magnitude = $\pi/5$ (as implemented; $\approx 36°$)
Translation	RandomAffine(degrees=0, translate=(0.2, 0.2))	Up to $\pm 20\%$ of image width/height in each axis
Rotation	RandomAffine(degrees=20)	Rotation angle $\theta \in [-20°,\ 20°]$
Fisheye distortion	fish(img, distortion_coefficient=0.2)	Distortion coefficient $k = 0.2$;
		$(c_x, c_y) \sim$ Uniform{48, … ,208} on a 224×224 grid

Table S1: Data augmentation parameter ranges. For augmented training samples, we decoded each image to grayscale and then applied one randomly chosen transformation from the set {scaling, shear, translation, rotation, fisheye} (via RandomChoice), followed by tensor conversion and intensity normalization.

Comment 5. Minor Correction – Table Formatting and Errors

Table 1 has inconsistent formatting. Table 3 lists "White" race AUC as 0.63 [0.61–0.65], but the confidence interval appears unusually narrow; please verify these values.

Response: We thank the reviewer for carefully checking the tabulated results. In the revised manuscript, we reviewed the formatting of the tables and corrected alignment and presentation issues for consistency and clarity.

We also rechecked the reported subgroup performance values and confidence intervals, including the White subgroup AUROC cited by the reviewer, to ensure that all entries reflect the final analysis outputs correctly. Any errors identified during this verification were corrected in the revised tables.

Comment 6. Minor Suggestion – Statistical Reporting
The Methods section mentions non-parametric bootstrap for CIs but does not specify the number of iterations or seed details. Add these parameters to enhance reproducibility. In Table 2, the "Intersection" group for "Worst group: White 80+ Male" has a wide CI; consider discussing the implications of high uncertainty in small subgroups.

Response: We appreciate this helpful comment. In the revised manuscript, we clarified the bootstrap procedure in the Methods section by explicitly stating the number of bootstrap resamples used to compute 95% confidence intervals and by clarifying the role of repeated training seeds in the model development workflow.

We also agree that wide confidence intervals in small intersectional subgroups are important to interpret explicitly. Accordingly, we revised the Results and Discussion to note that some intersectional subgroup estimates are based on limited sample sizes and should therefore be interpreted with caution. We now emphasize that these analyses are intended to highlight potentially important patterns of disparity while also illustrating the substantial uncertainty that can arise in small subpopulations.

Comment 7. Minor Clarification – Grad-CAM Interpretation
Figure 5 shows Grad-CAM heatmaps but lacks quantitative analysis of region importance. Supplement with metrics such as overlap with anatomical landmarks to strengthen claims of "biological plausibility."

Response: We thank the reviewer for this thoughtful suggestion. We agree that quantitative attribution analysis can be valuable in settings where a clinically meaningful signal is expected to localize consistently to a defined anatomical structure. However, in the present task, the discriminative signal separating HFrEF from HFpEF may reflect a combination of global or distributed radiographic patterns, rather than a single focal lesion or a fixed anatomical landmark. For this reason, we were concerned that introducing an anatomical-overlap metric could overstate localization precision and potentially imply a level of mechanistic specificity not supported by the task itself.

Instead, in the revised manuscript, we have taken two steps. First, we strengthened the methodological transparency of the Grad-CAM analysis by specifying the target layer, target output, and the across-seed stability procedure in both the main text and the figure caption.

Second, we revised the interpretation of these visualizations to emphasize that they provide qualitative and stability-oriented insight rather than quantitative evidence of biological localization. Our intention is therefore not to claim that the model consistently identifies a single anatomical substrate, but rather to show that the attribution patterns are not obviously unstable across random seeds and may reflect distributed image features relevant to the classification task.

Comment 8. Minor Edit – Reference Accuracy
Reference 4 (Rajpurkar et al.) is duplicated in the text as [4, 4–7]; correct to [4–7]. Reference 18 (Wang et al.) discusses augmentation but is not directly tied to the methods; clarify its relevance or cite more specific works.

Response: We thank the reviewer for identifying these citation issues. In the revised manuscript, we corrected the duplicated citation formatting and reviewed the augmentation-related references to ensure that they appropriately support the methodological statements in the text. Where needed, we clarified the relevance of the cited work and revised the reference presentation for accuracy and consistency.
