# Supplementary material for: Discriminating HFrEF vs HFpEF from chest radiographs: Mitigating demographic performance gaps via augmentation and multimodal fusion
Source: PLOS Digit Health. 2026 Jun 12;5(6):e0001467. doi: 10.1371/journal.pdig.0001467 (PMC13262823; doi:10.1371/journal.pdig.0001467)
Supplement: Peer review history

# Peer review: Decision Letter 0

**DOI:** 10.1371/journal.pdig.0001467.r001

**Authors:** Hyunjung Kwak

8 Jan 2026

PDIG-D-25-00730

Discriminating HFrEF vs HFpEF from chest radiographs: mitigating demographic performance gaps via augmentation and multimodal fusion

PLOS Digital Health

Dear Dr. Kuo,

Thank you for submitting your manuscript to PLOS Digital Health. After careful consideration, we feel that it has merit but does not fully meet PLOS Digital Health's publication criteria as it currently stands. Therefore, we invite you to submit a revised version of the manuscript that addresses the points raised during the review process.

Please submit your revised manuscript by Mar 09 2026 11:59PM. If you will need more time than this to complete your revisions, please reply to this message or contact the journal office at digitalhealth@plos.org. When you're ready to submit your revision, log on to https://www.editorialmanager.com/pdig/ and select the 'Submissions Needing Revision' folder to locate your manuscript file.

Please include the following items when submitting your revised manuscript:

* A letter that responds to each point raised by the editor and reviewer(s). You should upload this letter as a separate file labeled 'Response to Reviewers'. This file does not need to include responses to any formatting updates and technical items listed in the 'Journal Requirements' section below.

* A marked-up copy of your manuscript that highlights changes made to the original version. You should upload this as a separate file labeled 'Revised Manuscript with Track Changes'.

* An unmarked version of your revised paper without tracked changes. You should upload this as a separate file labeled 'Manuscript'.

If you would like to make changes to your financial disclosure, competing interests statement, or data availability statement, please make these updates within the submission form at the time of resubmission. Guidelines for resubmitting your figure files are available below the reviewer comments at the end of this letter.

We look forward to receiving your revised manuscript.

Kind regards,

Hyunjung Kwak

Section Editor

PLOS Digital Health

Hyunjung Kwak

Section Editor

PLOS Digital Health

Leo Anthony Celi

Editor-in-Chief

PLOS Digital Health

orcid.org/0000-0001-6712-6626

**Journal Requirements:**

1. We ask that a manuscript source file is provided at Revision. Please upload your manuscript file as a .doc, .docx, .rtf or .tex.

2. Please upload separate figure files in .tif or .eps format. Also, remove the figures from your manuscript file but keep the legends.

For more information about figure files please see our guidelines:

https://journals.plos.org/digitalhealth/s/figures

https://journals.plos.org/digitalhealth/s/figures#loc-file-requirements

3. We notice that your supplementary tables are included in the manuscript file. Please remove them and upload them with the file type 'Supporting Information'. Please ensure that each Supporting Information file has a legend listed in the manuscript after the references list.

4. Please note that your Data Availability Statement is currently missing [the repository name and/or the DOI/accession number of each dataset OR a direct link to access each database]. If your manuscript is accepted for publication, you will be asked to provide these details on a very short timeline. We therefore suggest that you provide this information now, though we will not hold up the peer review process if you are unable.

If the reviewer comments include a recommendation to cite specific previously published works, please review and evaluate these publications to determine whether they are relevant and should be cited. There is no requirement to cite these works unless the editor has indicated otherwise.

**Additional Editor Comments (if provided):**

[Note: HTML markup is below. Please do not edit.]

**Reviewers' Comments:**

Reviewer's Responses to Questions

**Comments to the Author**

1. Does this manuscript meet PLOS Digital Health’s publication criteria? Is the manuscript technically sound, and do the data support the conclusions? The manuscript must describe methodologically and ethically rigorous research with conclusions that are appropriately drawn based on the data presented.

Reviewer #1: Partly

Reviewer #2: Yes

**********

2. Has the statistical analysis been performed appropriately and rigorously?

Reviewer #1: Yes

Reviewer #2: Yes

**********

3. Have the authors made all data underlying the findings in their manuscript fully available (please refer to the Data Availability Statement at the start of the manuscript PDF file)?

The PLOS Data policy requires authors to make all data underlying the findings described in their manuscript fully available without restriction, with rare exception. The data should be provided as part of the manuscript or its supporting information, or deposited to a public repository. For example, in addition to summary statistics, the data points behind means, medians and variance measures should be available. If there are restrictions on publicly sharing data—e.g. participant privacy or use of data from a third party—those must be specified.

Reviewer #1: Yes

Reviewer #2: Yes

**********

4. Is the manuscript presented in an intelligible fashion and written in standard English?

PLOS Digital Health does not copyedit accepted manuscripts, so the language in submitted articles must be clear, correct, and unambiguous. Any typographical or grammatical errors should be corrected at revision, so please note any specific errors here.

Reviewer #1: Yes

Reviewer #2: Yes

**********

5. Review Comments to the Author

Please use the space provided to explain your answers to the questions above. You may also include additional comments for the author, including concerns about dual publication, research ethics, or publication ethics. (Please upload your review as an attachment if it exceeds 20,000 characters)

Reviewer #1: 1. Summary

This proof of concept study uses MIMIC-CXR images linked to MIMIC-IV ICD-10 labels (I50.2× vs I50.3×, restricted to CheXpert pulmonary edema positive cases) to distinguish HFrEF from HFpEF with a DenseNet-based model, evaluates data augmentation, and shows that a simple multimodal fusion of imaging with demographics and comorbidities increases AUC from approximately 0.64 to approximately 0.75 while narrowing demographic performance gaps.

2. Novelty issues

(1) The manuscript frames itself as a proof of concept showing that chest radiographs contain latent signal capable of separating HFrEF from HFpEF, and that fairness interventions such as data augmentation and multimodal fusion with demographic features (age, sex, self-reported race) can improve performance.

However, the core premise that chest radiographs encode EF-related signal has already been demonstrated at multi-institutional scale with external validation and echocardiographic ground truth. Ueda et al. (Lancet Digital Health, 2023) trained with patient-level splits using five-fold cross-validation across several institutions and evaluated on an independent external hospital; for LVEF less than 40 percent, the external test AUC was 0.92 (95% CI 0.90 to 0.95). That work also used echo-derived labels and reported broad cardiac targets (EF, valvular disease, TR velocity), thereby establishing more strongly than the present manuscript both the presence of EF information in chest radiographs and out-of-sample generalisability (DOI: 10.1016/S2589-7500(23)00107-3). If the novelty is articulated as showing that chest radiographs can separate EF phenotypes and that augmentation or multimodality improves performance, the contribution is materially diminished by the above evidence.

(2) A differentiated contribution can be claimed by reframing fairness as a first-class study aim. Unlike many high-performing CXR-EF studies, this manuscript explicitly targets demographic subgroup gaps and investigates interventions such as augmentation and fusion with demographic and comorbidity features to narrow them. The results section and figures foreground subgroup AUCs and Random Forest feature importance, in which race, age, and sex rank highest, which indicates that demographic context materially drives the fusion gains. This line of inquiry, namely systematic fairness analysis and disparity mitigation on a public dataset, can constitute the paper’s primary incremental value if it is presented clearly as such and supported with threshold-level metrics as outlined in section 3. A recommendation on positioning is to replace any first-of-its-kind framing about CXR-based EF discrimination with the following: “We provide a public-data, fairness-centred evaluation of CXR-clinical fusion for HF phenotype separation, and empirically show reduction of demographic performance gaps.” The manuscript should also acknowledge the performance and external-validity advantage of echo-labelled, multi-centre work such as Ueda et al. and the clinical-utility focus of Kusunose et al., and then delineate a distinct aim that emphasises equity-oriented methodology to avoid overclaiming novelty.

3. Experimental design and analysis

(1) Label definition and task boundary. The cohort is defined by ICD-10 codes (HFrEF and HFpEF) plus a CheXpert pulmonary edema label, which may bias toward acute congestion phenotypes rather than a pure EF phenotype; please report the timing windows among CXR, ICD, and edema labels, and discuss differences from echo-labelled tasks, for example Ueda et al.

(2) Unit of split and potential leakage. The dataset comprises 3,853 unique admissions, and a single 70/21/9 partition is used; it is not explicit that splitting is at the patient level. Please enforce patient-level splits and report both admission-level and patient-level results.

(3) Fairness evidence at decision thresholds. Current claims rely mainly on changes in AUC gaps. Please add threshold-level subgroup metrics, such as FPR, FNR, PPV, and NPV at a common sensitivity or a common threshold, with confidence intervals, and include calibration measures such as Brier score and calibration slope and intercept overall and by subgroup.

(4) Use of sensitive features. Given that race, age, and sex dominate Random Forest feature importance, please include a sensitivity analysis that removes race from the fusion inputs and discuss the associated ethical trade-offs.

4.Minor issues

Duplicate word: “collected from from the Beth Israel…” → remove one “from”.

Terminology: “translate transformation” → “translation (transformation)”.

Punctuation: “,These biases may arise …” → remove the comma after the opening punctuation.

Figure narration: Grad-CAM is shown; add a sentence on settings and stability checks, such as the averaging protocol, in the caption or the supplement.

5.Overall recommendation: Major revision. As currently written, the manuscript’s novelty is limited relative to multi-centre, externally validated, echo-labelled work on CXR-based EF and related targets, such as Ueda et al., and relative to studies demonstrating incremental clinical utility, such as Kusunose et al. The paper’s most defensible contribution is a fairness-centred evaluation and mitigation program on a public dataset. Repositioning the contribution accordingly and adding patient-level splits, threshold-level subgroup metrics and calibration, and a race-feature sensitivity analysis would substantially improve clarity, methodological rigour, and the strength of the claims.

Reviewer #2: Summary

This manuscript presents a proof-of-concept study investigating the use of deep learning models to differentiate between heart failure with reduced ejection fraction (HFrEF) and heart failure with preserved ejection fraction (HFpEF) from chest radiographs. The authors employ data augmentation and multimodal fusion with demographic features to improve model performance and address demographic performance gaps. Key findings include an AUC improvement from 0.64 with augmentation to 0.75 with multimodal fusion, along with substantial reductions in race- and sex-based performance disparities. The study utilizes the MIMIC-CXR dataset, DenseNet121 architecture, and fairness evaluation metrics, supported by Grad-CAM visualizations. While the results are promising for equitable AI applications in resource-limited settings, several methodological and clinical considerations warrant attention.

Major Comments

Major Concern - Clinical Validation and Generalizability

The cohort selection criteria (lines referencing Fig. 1) focused on patients with acute HF presentations (defined by pulmonary edema on CXR and new ICD-10 codes), which may limit the model's applicability to chronic HF populations. The Discussion section (paragraph 4) acknowledges this but does not provide stratified analyses. To strengthen clinical relevance, the authors should either:

a) Conduct subgroup analyses separating acute versus chronic HF cases, or

b) Explicitly state this limitation in the Abstract and Conclusions with recommendations for future validation in diverse clinical settings.

Major Concern - Modest Model Performance

The overall AUC of 0.75 for the multimodal model (Table 4), while improved, remains modest for clinical decision-making. The 95% CIs for some subgroups (e.g., Black patients: AUC 0.71 [0.67-0.75]) suggest overlapping performance, raising questions about robustness. The authors should:

a) Discuss the clinical implications of these AUC values in the context of existing diagnostic tools (e.g., echocardiography),

b) Provide sensitivity and specificity metrics to aid interpretability, and

c) Consider external validation to confirm generalizability.

Major Concern - Fairness Evaluation Methodology

The fairness analysis measured performance gaps (e.g., Black vs. White AUC difference) but did not employ established fairness metrics (e.g., equalized odds, demographic parity). The Results section (Tables 2-4) reports disparities but lacks statistical tests for significance of gap reductions. To enhance rigor, the authors should:

a) Incorporate standardized fairness metrics and statistical comparisons of disparity changes,

b) Justify the focus on race, age, and sex without addressing other potential biases (e.g., socioeconomic status), and

c) Include sample sizes for subgroups to assess power limitations.

Major Concern - Data Augmentation Impact

Data augmentation improved equity but the choice of techniques (rotation, shear, etc.) was not justified based on CXR-specific characteristics. The Methods section ("Data Augmentation") states parameters were "randomly selected," which may introduce noise. The authors should:

a) Provide rationale for selecting these augmentations over others (e.g., contrast adjustments common in medical imaging),

b) Report the magnitude ranges used for each transformation, and

c) Discuss whether augmentations could artificially alter clinically relevant features (e.g., cardiac silhouette distortion).

Minor Comments

Minor Correction - Table Formatting and Errors

Table 1 has inconsistent formatting (e.g., "HFrEF" column, row for "Imaging location" has misaligned percentages). Please verify and align all entries for clarity.

Table 3 lists "White" race AUC as 0.63 [0.61-0.65], but the confidence interval appears unusually narrow; check for calculation or reporting errors.

Minor Suggestion - Statistical Reporting

The Methods section mentions "non-parametric bootstrap" for CIs but does not specify the number of iterations or seed details. Add these parameters to enhance reproducibility.

In Table 2, the "Intersection" group for "Worst group: White 80+ Male" has an AUC of 0.43 with a wide CI [0.27-0.60]; consider discussing the implications of high uncertainty in small subgroups.

Minor Clarification - Grad-CAM Interpretation

Figure 5 shows Grad-CAM heatmaps but lacks quantitative analysis of region importance. Supplement with metrics (e.g., overlap with anatomical landmarks) to strengthen claims of "biological plausibility" (Discussion, paragraph 1).

Minor Edit - Reference Accuracy

Reference 4 (Rajpurkar et al.) is duplicated in the text as [4, 4–7]; correct to [4–7] for accuracy.

Reference 18 (Wang et al.) discusses augmentation but is not directly tied to the methods; clarify its relevance or cite more specific works.

Recommendations

Based on the review, I recommend major revision prior to further consideration. The study addresses an important gap in equitable AI for HF diagnosis but requires:

Enhanced methodological rigor in fairness evaluation and statistical reporting.

Deeper discussion of clinical and ethical implications, particularly regarding demographic data use.

Additional analyses to address generalizability concerns (e.g., acute vs. chronic HF stratification).

With these revisions, the manuscript has the potential to contribute significantly to the field of digital health and algorithmic fairness.

**********

6. PLOS authors have the option to publish the peer review history of their article (what does this mean?). If published, this will include your full peer review and any attached files.

**Do you want your identity to be public for this peer review?**  If you choose “no”, your identity will remain anonymous but your review may still be made public.

For information about this choice, including consent withdrawal, please see our Privacy Policy.

Reviewer #1: No

Reviewer #2: No

**********

[NOTE: If reviewer comments were submitted as an attachment file, they will be attached to this email and accessible via the submission site. Please log into your account, locate the manuscript record, and check for the action link "View Attachments". If this link does not appear, there are no attachment files.]

**Figure resubmission:**

While revising your submission, we strongly recommend that you use PLOS’s NAAS tool (https://ngplosjournals.pagemajik.ai/artanalysis) to test your figure files. NAAS can convert your figure files to the TIFF file type and meet basic requirements (such as print size, resolution), or provide you with a report on issues that do not meet our requirements and that NAAS cannot fix.

After uploading your figures to PLOS’s NAAS tool - https://ngplosjournals.pagemajik.ai/artanalysis, NAAS will process the files provided and display the results in the "Uploaded Files" section of the page as the processing is complete. If the uploaded figures meet our requirements (or NAAS is able to fix the files to meet our requirements), the figure will be marked as "fixed" above. If NAAS is unable to fix the files, a red "failed" label will appear above. When NAAS has confirmed that the figure files meet our requirements, please download the file via the download option, and include these NAAS processed figure files when submitting your revised manuscript.

**Reproducibility:**

To enhance the reproducibility of your results, we recommend that authors of applicable studies deposit laboratory protocols in protocols.io, where a protocol can be assigned its own identifier (DOI) such that it can be cited independently in the future. Additionally, PLOS ONE offers an option to publish peer-reviewed clinical study protocols. Read more information on sharing protocols at https://plos.org/protocols?utm_medium=editorial-email&utm_source=authorletters&utm_campaign=protocols

# Author comment: Author response to Decision Letter 1

**DOI:** 10.1371/journal.pdig.0001467.r002

10 Mar 2026

Attachment Submitted filename: Response letter.pdf

# Peer review: Decision Letter 1

**DOI:** 10.1371/journal.pdig.0001467.r003

**Authors:** Matthew Watson

12 May 2026

Discriminating HFrEF vs HFpEF from chest radiographs: mitigating demographic performance gaps via augmentation and multimodal fusion

PDIG-D-25-00730R1

Dear Professor Kuo,

We are pleased to inform you that your manuscript 'Discriminating HFrEF vs HFpEF from chest radiographs: mitigating demographic performance gaps via augmentation and multimodal fusion' has been provisionally accepted for publication in PLOS Digital Health.

Before your manuscript can be formally accepted you will need to complete some formatting changes, which you will receive in a follow-up email from a member of our team.

Please note that your manuscript will not be scheduled for publication until you have made the required changes, so a swift response is appreciated.

IMPORTANT: The editorial review process is now complete. PLOS will only permit corrections to spelling, formatting or significant scientific errors from this point onwards. Requests for major changes, or any which affect the scientific understanding of your work, will cause delays to the publication date of your manuscript.

If your institution or institutions have a press office, please notify them about your upcoming paper to help maximize its impact. If they'll be preparing press materials, please inform our press team as soon as possible -- no later than 48 hours after receiving the formal acceptance. Your manuscript will remain under strict press embargo until 2 pm Eastern Time on the date of publication. For more information, please contact digitalhealth@plos.org.

Thank you again for supporting Open Access publishing; we are looking forward to publishing your work in PLOS Digital Health.

Best regards,

Matthew Watson

Guest Editor

PLOS Digital Health

***********************************************************

**Additional Editor Comments (if provided):**

Many thanks for all of the work that went into addressing reviewer comments on this manuscript. All comments have been addressed and this is a strong paper that has now been accepted for publication. Congratulations!

**Reviewer Comments (if any, and for reference):**

Reviewer's Responses to Questions

**Comments to the Author**

1. If the authors have adequately addressed your comments raised in a previous round of review and you feel that this manuscript is now acceptable for publication, you may indicate that here to bypass the “Comments to the Author” section, enter your conflict of interest statement in the “Confidential to Editor” section, and submit your "Accept" recommendation.

Reviewer #2: All comments have been addressed

**********

2. Does this manuscript meet PLOS Digital Health’s publication criteria? Is the manuscript technically sound, and do the data support the conclusions? The manuscript must describe methodologically and ethically rigorous research with conclusions that are appropriately drawn based on the data presented.

Reviewer #2: Yes

**********

3. Has the statistical analysis been performed appropriately and rigorously?

Reviewer #2: Yes

**********

4. Have the authors made all data underlying the findings in their manuscript fully available (please refer to the Data Availability Statement at the start of the manuscript PDF file)?

The PLOS Data policy requires authors to make all data underlying the findings described in their manuscript fully available without restriction, with rare exception. The data should be provided as part of the manuscript or its supporting information, or deposited to a public repository. For example, in addition to summary statistics, the data points behind means, medians and variance measures should be available. If there are restrictions on publicly sharing data—e.g. participant privacy or use of data from a third party—those must be specified.

Reviewer #2: Yes

**********

5. Is the manuscript presented in an intelligible fashion and written in standard English?

PLOS Digital Health does not copyedit accepted manuscripts, so the language in submitted articles must be clear, correct, and unambiguous. Any typographical or grammatical errors should be corrected at revision, so please note any specific errors here.

Reviewer #2: Yes

**********

6. Review Comments to the Author

Please use the space provided to explain your answers to the questions above. You may also include additional comments for the author, including concerns about dual publication, research ethics, or publication ethics. (Please upload your review as an attachment if it exceeds 20,000 characters)

Reviewer #2: None.

**********

7. PLOS authors have the option to publish the peer review history of their article (what does this mean?). If published, this will include your full peer review and any attached files.

**Do you want your identity to be public for this peer review?**  If you choose “no”, your identity will remain anonymous but your review may still be made public.

For information about this choice, including consent withdrawal, please see our Privacy Policy.

Reviewer #2: No

**********
